# The Future of Virology is Synthetic

**DOI:** 10.1128/mSystems.00770-21

**Published:** 2021-08-31

**Authors:** Richard Allen White

**Affiliations:** a Department of Bioinformatics and Genomics, The University of North Carolina at Charlotte, Charlotte, North Carolina, USA; b Department of Bioinformatics and Genomics, The University of North Carolina at Charlotte, Kannapolis, North Carolina, USA; c Australian Centre for Astrobiology, University of New South Wales Sydney, Sydney, Australia

**Keywords:** viruses, bacteriophage (phage), mycovirus, multidrug-resistant microbes, virosphere, Hendrix product, viral auxiliary metabolic genes (vAMGs), massive parallel sequencing (MPS), synthetic biology, rules of life, silent pandemic, climate change, engineering, sustainable agriculture

## Abstract

The virosphere (i.e., global virome) represents a vast library of unknown genes on the planet. Synthetic biology through engineering principles could be the key to unlocking this massive global gene repository. Synthetic viruses may also be used as tools to understand “the rules of life” in diverse microbial ecosystems. Such insights may be crucial for understanding the assembly, diversity, structure, and scale of virus-mediated function. Viruses directly affect resilience, stability, and microbial community selection via death resistance cycles. Interpreting and clarifying these effects is essential for predicting the system’s ecology, evolution, and ecosystem stability in an increasingly unstable global climate. A “silent looming pandemic” due to multidrug-resistant microbes will directly impact the global economy, and synthetic virology could provide a future strategy of treatment using targeted viral therapy. This commentary will discuss current techniques for manipulating viruses synthetically, contributing to improved human health and sustainable agriculture.

## COMMENTARY

## WHAT IS SYNTHETIC VIROLOGY?

**FIG 1:**
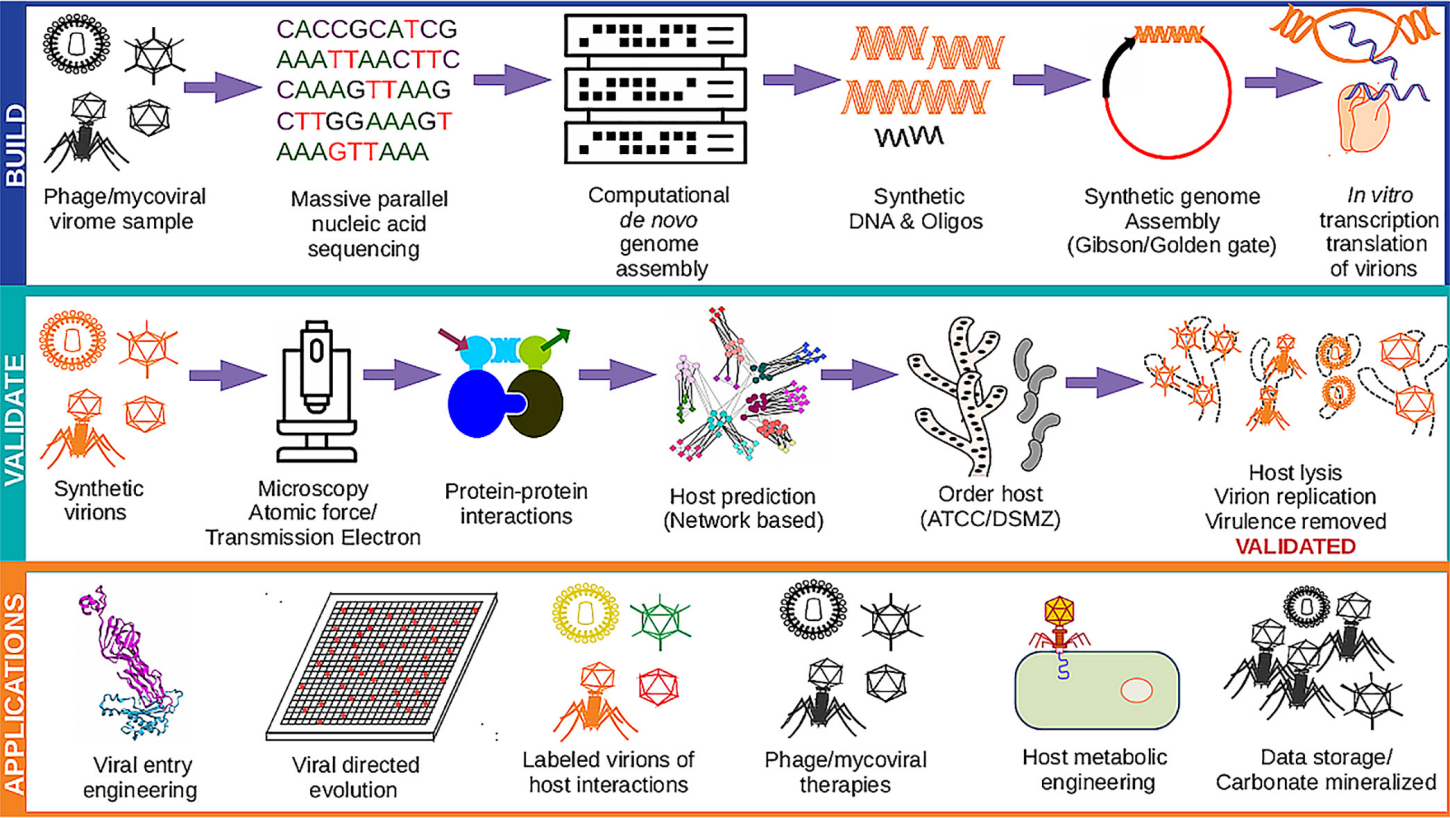
Building synthetic viruses from start to finish, including applications. (Top [blue]) Process of building synthetic viruses. First, viral nucleic acids must be extracted and then sequenced using massively parallel nucleic acid sequencing. After sequencing, computational pipelines assemble the viral genomes *de novo*. Once the viral genomes are assembled computationally, synthetic DNA and oligonucleotides (oligos) can be ordered. Next, the synthetic DNA and oligos can be assembled into full-length viral genomes using Gibson or Golden Gate assembly. Finally, the assembled viral genome can be converted into viral particles using *in vitro* transcription and translation into synthetic virions. (Middle [teal]) How to validate synthetic virions. Microscopy, either atomic force or transmission electron microscopy, should be used to validate the presence of virions. Protein-protein interactions with complete or incomplete virions can be used to measure host-viral interactions. Hosts can be predicted computationally utilizing a variety of methods (3). If the host is currently available, it can be ordered and then validated for virion replication, host lysis, or removal of host virulence genes. (Bottom [orange]) Variety of applications for synthetic virions. These applications range from viral nucleic acid data storage and mineralizing virions into carbonates to phage/mycoviral therapies.

Synthetic virology is a subdiscipline of virology that applies molecular, computational, and synthetic biology principles from the fundamentals obtained from naturally occurring viruses to engineer viruses. The first virus assembled from synthetic oligonucleotides was poliovirus (1), followed by the phiX174 bacteriophage (i.e., phage) (2). Synthetic viruses are built upon a previously sequenced genome, and then oligonucleotides are ordered and assembled synthetically (e.g., Gibson) (2). Synthetic virions should be evaluated morphologically using transmission electron microscopy (TEM) or atomic force microscopy (AFM) to ensure viral genome packaging within particles (i.e., complete virion assembly) before host validation as a quality control step. Computational tools can predict hosts of various viruses via a variety of methods (3). If the host is available, an infection could be confirmed from a synthetic virus. If successful, it would enable a user to construct model systems directly from sequencing data (Fig. 1).

In the early 1950s, it was unknown what molecule drove heredity, whether it was nucleic acids or protein. Finally, Hershey and Chase (4), using T2 phage, confirmed it was DNA, using synthetic radiolabeled phage proteins (^35^S) and DNA (^32^P). Thus, viruses established the first rule of life that nucleic acids, not protein, was the molecule of inheritance. Therefore, synthetic viruses were used to establish the first rule of life; now, viruses can be used to understand the assembly, diversity, structure, and scale of virus-mediated influence.

Synthetic biology represents a significant opportunity for economic advancement, including an estimated $11.4 billion market by 2021 (5). Synthetic viruses could even be engineered to perform specific tasks and may have broad applications in agriculture, medicine, climate change, and, potentially, carbon capture (Fig. 1).

## ESTIMATING THE LARGEST GENETIC REPOSITORY ON EARTH—THE VIROSPHERE

The virosphere, which is a collection of all of Earth’s viruses, represents the most abundant biological entities (6, 7). The virosphere is estimated at an abundance 10^31^ virus-like particles (VLPs) called the “Hendrix product.” (6). This Hendrix product is larger than a mole of atoms (6.022 × 10^23^, Avogadro’s number), more numerous than stars in the observable universe (10^21^) and greater than the number of all the cells in the human body (10^13^) (6–8). The human body also contains a vast abundance of viruses. The oral virome is ∼10^11^ VLPs (assuming 10^8^ ml^−1^ in 1.5 liter of saliva), the stool virome is ∼4.5 × 10^11^ VLPs (assuming 10^9^ g^−1^ in 454 g of stool), and the urine virome is 7 × 10^9^ VLPs (assuming 10^7^ g^−1^ in 700 ml of urine) (9, 10). The numbers of VLPs in stool and oral viromes, at ∼10^11^, are equivalent to the number of stars in the Milky Way galaxy. A diverse virome is interacting through life and death struggles daily within the human body.

Viral abundances can be measured directly via staining nucleic acids or indirectly via measuring nucleic acids. For indirect measurement, *a priori* information is required about the viral genome, and then a PCR-based diagnostic (e.g., digital PCR) (11) can be used to estimate viral genome equivalents. A random nucleic acid stain is used (e.g., SYBR), and then VLPs are counted with epifluorescence microscopy (12) or flow cytometry (13). Dyes such as SYBR are double-stranded DNA (dsDNA) specific and fail to stain single-stranded DNA (ssDNA) and ssRNA effectively (7). Giant viruses are filtered out and thus rarely counted (7). A single RNA virus pandemic (e.g. severe acute respiratory syndrome coronavirus 2 [SARS-CoV-2]) can reach numbers >10^17^ (14); hence, the Hendrix product must be larger to account for giant viruses and RNA and ssDNA viruses. Advances in viral counting are needed, including (i) measurement of intact particles, (ii) new dyes to differentiate nucleic acid types and strand types (RNA versus DNA, single versus double), (iii) particle size measurement, and (iv) an increase in overall throughput. AFM and TEM must be further explored for viral abundance measurements.

Massive parallel sequencing (MPS; formally NextGen) has elucidated vast numbers of new viral genomes; however, we cannot unlock the functions of the enormous library of unknown genes. We currently struggle to provide functional gene validation to even highly studied organisms such as Escherichia coli, in which 35% of the genome has no functional validation (15). The virosphere harbors an immense gene repertoire of ∼10^32^ genes (if we assume ∼10 genes per virus with the Hendrix product). Viruses fair worse than E. coli in functional validation, with ∼50% to 70% of the genes lacking functional validation. Such numbers, if validated, would provide a massive library of genes for synthetic biology.

## VIRUSES TO TACKLE CLIMATE CHANGE WHILE PROVIDING SUSTAINABLE AGRICULTURE

The impact of viruses on global biogeochemical cycles is noted and described broadly elsewhere (16). The roles of viruses manipulating biogeochemical cycling have been documented via viral auxiliary metabolic genes (vAMGs) (16). vAMGs are involved in many biogeochemical processes, from photosynthesis to carbon and phosphorus metabolism (16).

Photosynthetic engineering could increase carbon capture and storage within the lithosphere, relieving the climate crisis via photosynthesis-induced alkalinity to precipitate carbonate minerals (17). Viruses carrying vAMGs can remodel carbon metabolism in cyanobacteria (16), which could be further engineered with synthetic viruses to increase carbonate precipitation. Viral lysis of cyanobacteria induces calcium carbonate mineral precipitation (7). Engineering viruses via direct lysis or via vAMG could enhance this process, leading to long-term carbon capture within minerals. Synthetic viruses themselves could be mineralized and then trap carbon on geological time scales.

How vAMGs partition microbial metabolisms within the terrestrial ecosystems, including the rhizosphere relating to carbon, nitrogen, phosphorus, and plant productivity, is unknown. Phages carry a vAMG homolog to *phoH* (phosphate starvation-inducible protein) and *pstS* (phosphate-binding protein), both activated under phosphorus starvation (16). While the function of *phoH* and *pstS* in phages is unknown, should these vAMGs be shown to enhance microbial metabolism related to phosphorus, this could provide plant growth-promoting effects. Therefore, viruses should be screened for plant growth-promoting processes beyond pathogen control within the rhizosphere microbiome.

Engineered viruses could be trained to eliminate pesticides, antibiotics, and fungicides. In addition, viral cocktails could provide a targeted treatment directly toward a pathogen within the field or feedlot. These viral cocktails could be designed to kill, reprogrammed to remove pathogen virulence genes, or made to push cells into dormancy.

## TEACHING AN OLD TECHNOLOGY NEW TRICKS TO TACKLE THE “SILENT PANDEMIC”

WHO predicts that 10 million people will die from a drug-resistant microbial infection (DRMI) by 2050 (18). Currently, at least 700,000 people die each year from DRMIs (18). We are running out of drugs for microbial resistance, for which many now have no treatment, causing a “silent pandemic.” Antibiotics are classically used as growth promoters in feedstocks (e.g., chickens, pigs, etc.) but increase DRMIs in a variety of bacterial pathogens (e.g., E. coli and Staphylococcus aureus) (19). Because antibiotics are bacteriostatic (nonlethal), microbes can escape via resistant mutations. The significant use of antibiotics in agriculture and medical overuse for nonbacterial infection increase the likelihood of DRMI spread.

Phage therapy is not a new idea; Felix d'Herelle proposed it at the beginning of the 20th century. Phage therapy offers targeted bactericidal (lethal) treatment for bacteria. Phage counterparts, mycoviruses, have been used as biocontrol agents to treat fungal infections (20). As with phage, mycoviruses could be engineered for better biocontrol of fungal pathogens. As mentioned above, they could be designed, engineered, and tailored as cocktails for a specific pathogen. Also, viruses could be combined with antibiotics to make them effective again by adding back antibiotic-sensitive genes (21) or via an evolutionary approach (22). Evolutionary approaches to phage therapy combined with antibiotics are modeled and described elsewhere (22).

Questions remain about the use of phage and mycoviral therapy in human patients, including safety, resistance, specificity, and biofilm penetration. Phages can degrade biofilms via depolymerase (23), which can be engineered to be highly specific. The benefit of phage therapy, especially if tailored toward specific pathogens, is that it could be effective and safe, even for immunocompromised patients; however, further clinical studies are needed (24). Less is known on mycoviral therapy for patients, as only viral-like particles, but no viruses, have been isolated for common human fungal pathogens, including Candida albicans (20). In addition, bacteria and fungi can develop resistance to viral therapy; however, using multiviral cocktails of >5 viruses or more limits this resistance (25).

Viruses are commonly very specific to single hosts; however, they can be polyvirulent and infect many members of the same species. It has not been widely observed that viruses jump into different phyla, and they rarely jump beyond the class of their original host. The main issue is specificity, which can be combated by cocktail design or viral entry engineering.

## CONCLUSION

Viruses have been the catalysts for molecular biology, synthetic biology, and the genome sequencing revolution. Viruses have elucidated cellular mechanisms essential to regulating our microbiome and are fundamental to Earth’s carbon cycle. Viral genes should be used creatively in research; to solve your questions, learn from them and use these technologies to solve global issues ranging from climate change to the silent pandemic. Viral-based mechanisms (e.g., CRISPR) can be disruptive technologies that are massive innovations. While the COVID-19 pandemic has highlighted the pivotal role of viruses in our daily life, remember that viruses are more friend than foe, and they are the future via synthetic biology.
